# Influence of Soil Properties on Soldierless Termite Distribution

**DOI:** 10.1371/journal.pone.0135341

**Published:** 2015-08-13

**Authors:** Thomas Bourguignon, Thomas Drouet, Jan Šobotník, Robert Hanus, Yves Roisin

**Affiliations:** 1 Department of Biological Sciences, National University of Singapore, 117543, Singapore, Singapore; 2 Czech University of Life Sciences, Faculty of Forestry and Wood Sciences, Kamýcká 129, 165 21, Praha 6 –Suchdol, Czech Republic; 3 Plant Ecology and Biogeochemistry, Université Libre de Bruxelles, Brussels, Belgium; 4 Institute of Organic Chemistry and Biochemistry, Flemingovo nám. 2, 166 10, Prague, Czech Republic; 5 Evolutionary Biology and Ecology, CP 160/12, Université Libre de Bruxelles, Avenue F.D. Roosevelt 50, 1050, Brussels, Belgium; Universidade de São Paulo, BRAZIL

## Abstract

In tropical rainforests, termites constitute an important part of the soil fauna biomass, and as for other soil arthropods, variations in soil composition create opportunities for niche partitioning. The aim of this study was twofold: first, we tested whether soil-feeding termite species differ in the foraging substrate; second, we investigated whether soil-feeding termites select their foraging sites to enhance nutrients intake. To do so, we collected termites and analysed the composition and structure of their feeding substrates. Although *Anoplotermes*-group members are all considered soil-feeders, our results show that some species specifically feed on abandoned termite nests and very rotten wood, and that this substrate selection is correlated with previous stable isotope analyses, suggesting that one component of niche differentiation among species is substrate selection. Our results show that the composition and structure of bare soils on which different termite species foraged do not differ, suggesting that there is no species specialization for a particular type of bare soil. Finally, the bare soil on which termites forage does not differ from random soil samples. Overall, our results suggest that few species of the *Anoplotermes*-group are specialized toward substrates rich in organic matter, but that the vast majority forage on soil independently of its structural and chemical composition, being ecologically equivalent for this factor.

## Introduction

Soil is one of the most complex and species-rich habitats, hosting a wide range of life forms [1]. A single square metre of soil can host over a thousand invertebrate species [2], raising questions about how such a large number of species coexist in this environment. Two non-exclusive, scale-dependent processes, can account for this high species diversity: stochastic population drift as predicted by neutral theory and niche partitioning mediated by environmental filtering [3]. Neutral theory has rarely been investigated for soil organisms, although recent studies on oribatid mite and springtail assemblages suggested it plays only a minor role in shaping communities [4, 5]. The study of stochastic processes linked with soil species diversity is still in its infancy. Despite looking homogeneous, soil might have a high niche dimensionality, allowing niche partitioning, time or spatial staggering [6]. Many soil arthropods are not generalists, but are instead specialized on particular ecological conditions, although the extent of this specialization is not completely understood yet. Specialization includes trophic niche differentiation (oribatid mites [7, 8]; springtails [9, 10]), specialisation to soil type, pH or nutrient availability [11, 12], which together with soil spatial heterogeneity provide a major explanatory factor for the high biodiversity in soil communities [6, 13].

Among the factors affecting soil fauna, nutrient availability plays a major role. Sodium is a factor limiting ant and termite abundances in terrestrial ecosystems [14, 15]; by favouring litter decomposing microorganisms, the addition of nitrogen and phosphorus promotes activity of many taxa from the mesofauna, but also results in a reduction in the standing litter volume, which negatively affects some taxa such as predatory ants [16]. Other components such as the organic matter accumulated at the base of palm trees or under bromeliad rosettes also have a positive effect on the abundance of arthropods such as termites [17, 18].

Termites are extremely abundant in many tropical ecosystems. Termites form two fundamental ecological groups differing in their diet, the soil-feeders (comprising only Termitidae species) and the non-soil-feeders (present in all termite families), whose diet is based on wood, grass, leaf-litter or microepiphytes. Besides their fundamental role as direct decomposers of plant matter, often ingesting the majority of the dead plant tissues in the tropics [19], they also frequently dominate the soil macrofauna in terms of abundance and biomass [20, 21, 22]. Additionally, they are often referred to as ecosystem engineers because they shape the environment through their action, considerably influencing parameters such as soil granulometry and chemical composition, or water retention, and strongly affecting animal and plant distribution [23]. The *Anoplotermes*-group is one of the most diversified termite taxa in South American rainforests, often represented by over 30 species in a single site [17, 24].

The *Anoplotermes*-group members are known to feed on soil, or at the wood-soil interface, with the exception of *Ruptitermes* which feeds on leaf litter [25, 26]. The precise diet of soil-feeding termites is only known for African species of the *Cubitermes* group (Cubitermitinae): their workers ingest argilo-humic compounds from which they digest the peptidic fraction, released through alkaline hydrolysis [27, 28, 29]. Whether *Anoplotermes*-group species also feed on the peptidic fraction of soil is unclear because the gut structure of their workers substantially differs from that of *Cubitermes* and related genera, suggesting functional differences. Additionally, the distribution of ^15^N:^14^N ratios within the *Anoplotermes* species group suggests a gradient of preferences for the degree of decomposition of the organic matter ingested [30], perhaps indicative of competitive displacements [31]. In this study we sampled foraging termites and the soil immediately adjacent to the specimens and tested for an association between species presence and a set of 27 soil parameters. Our study made no *a priori* assumptions about the feeding-group of the species [25] and sampled all foraging parties of *Anoplotermes*-group members without distinction. We aimed at answering two questions:

1. Do soil-feeding termite species differ in the foraging substrate?
2. Do soil-feeding termites select their foraging sites to enhance nutrients intake?

## Materials and Methods

### Study Site

Fieldwork took place in the Nouragues Nature Reserve, French Guiana (N 04°05’, W 52°41’) between 12 and 26 January 2010. The site experiences about 3000mm of rainfall per year, mostly distributed between January and June and with a drier season from September to November. The mean annual temperature is 26°C. The Nouragues is uninhabited and almost completely free from human disturbances.

In order to maximise the diversity of sampled habitats, we collected termites in 10 parcels of 1ha around the station. The plots were scattered on the slope of the Nouragues granite outcrop, from topmost rocky savannah to flat riverbank. The substrates exploited by termites were variable and included: poor aggregations of organic matter bellow stones in the rocky savannah (leptosols), richer soils (dystric nitisols), and organic matter-rich substrates represented by abandoned nests. We also sampled rotten wood or organic matter accumulations below trees, and poor sandy soils repeatedly washed by the river during seasonal floods (dystric nitisols with gleyic properties).

Parcels A-C and E-G were lowland primary rainforest growing on well-drained soil. Parcel D was very similar to the former six, but differed in the high density of lianas. Parcel H consisted of a forest growing beside a creek on poorly drained soil. Parcel J was a low forest growing on the foothills of the Nouragues inselberg, transitional between lowland rainforest and the rock savannah growing on the granitic outcrop. Finally, parcel I was located at the frontier of a low forest and a typical primary lowland rainforest, representing an intermediate type between the two types [32].

### Ethics Statement

The research permits required to work in the Nouragues Nature Reserve were included in the Nouragues project we got from Le Centre national de la recherche scientifique (CNRS). Our work did not involve any endangered or protected species.

### Sampling

Sampling effort was about three men/day for each parcel and did not follow a systematic protocol. We instead searched for termites of the *Anoplotermes*-group in all suitable microhabitats, namely: in the soil, in rotten pieces of wood or in abandoned termite nests. Six types of samples were collected: five categories of foraging substrates and one category of controls, i.e., soil samples without termite activity. Three controls were collected in each parcel, always from bare ground selected randomly for their absence of any termite activity. The five types of foraging substrates were: (1) abandoned nest = abandoned material from termite nests, devoid of its original builders, usually built by *Labiotermes labralis* and sometimes possibly by *Embiratermes neotenicus*, but often modified by other termite species; (2) rotten wood = pieces of decayed wood on which termites were feeding; (3) bare soil = soil not taken from near the base of any tree, palm or other type of vegetation; (4) soil (palm) = soil situated at the base of a palm tree; (5) soil (tree) = soil situated at the base of a tree, generally between buttress roots. For each foraging party encountered, we collected a voucher sample composed of 20 to 50 termite workers in 80% alcohol for species identification, recorded the type of substrate and sampled the foraging substrate for subsequent analyses. Species identification was carried out based on worker morphology and DNA barcoding [32]. Foraging substrate was collected right at the place where foraging termites were found. To ensure that samples were foraging parties and not galleries, only samples composed of more than 20 individuals were collected. 50 to 100g of soil or abandoned nest material were stored at -20°C, dried at 50°C and shipped to Belgium where analyses were carried out. Altogether, the foraging substrate of 173 termite parties was recorded, and 118 samples of termites were collected with their associated foraging substrate (S1 Table). Dead wood was not analysed in the present work because it is fully organic and cannot be compared to soil in many aspects such as its texture. Thirty additional control soil samples were analysed.

### Soil Analyses

Twenty-seven physico-chemical variables were measured on each soil sample: total carbon and nitrogen, exchangeable cations, exchangeable acidity, available phosphorus, pH (H_2_O and KCl extracts), organic matter content and soil texture (clay, silt and sand).

Soil analyses were carried out following standard protocols [33]. Soil samples were air-dried and sieved at 2 mm. Total carbon (C) and nitrogen (N) content was determined on crushed samples with a dry combustion C-N analyzer. Exchangeable cations and plant available phosphorus were extracted with 1 M ammonium acetate EDTA at pH 4.65 and measured with ICP-OES (Ca^2+^, Mg^2+^, K^+^, Fe^3+^, Zn^2+^, Cd^2+^, Pb^2+^, Cu^2+^, Ni^2+^). Exchangeable acidity and exchangeable aluminium were extracted with 1 M KCl and determined by derivative titration curves (H^+^ and Al^3+^). The cation exchange capacity (CEC) was calculated as the sum of base cations and the exchangeable acidity expressed in cmol_c_ kg^-1^. Soil pH-H_2_O and KCl were measured using a combination of glass electrodes in a 1:4 (v/v) slurry after 2 h of contact. Organic matter content (OM) was determined by loss on ignition at 550°C. Soil texture (i.e. proportions of clay, silt and sand) was determined using 10 g of sample after H_2_O_2_ pre-treatment and dispersion with Na citrate. The sand fraction was separated by wet sieving, and the clay and silt fractions were determined using a sedimentation method.

### Data Analyses

We used a detrended correspondence analysis (DCA) to visualise species association with type of substrate. The analysis was carried out using the CANOCO software with the option detrending by segment, using 26 segments, that allows rescaling each segment to mean value of zero on the second axis [34]. Species that were encountered on fewer than four occasions were discarded from the analysis as the detection of species-site association is dubious if it is based on too few records. The correlation between species scores along the first DCA axis and the mean δ^15^N isotopic values previously measured for 14 species [30] was investigated using Pearson correlation (note that the δ^15^N isotopic values were obtained from different samples). Differences between feeding substrates (abandoned nest, bare soil, soil (palm) and soil (tree)) and control were investigated using Kruskal-Wallis tests on each soil variable separately, testing for differences between substrate types. Multiple comparisons of mean ranks, implemented in Statistica, were used to test for differences between groups. Dunn-Šidák corrections were used to avoid errors resulting from multiple comparisons. To investigate differences between species in terms of feeding substrate composition, Kruskal-Wallis tests followed by multiple comparisons of mean ranks and Dunn-Šidák corrections were computed using each soil variable separately for all species with four measurements or more, and only from bare soil samples. We computed a principal component analysis (PCA) in order to dimensionally reduce the dataset and facilitate its visualization. We used only the samples for which the complete series of soil variables measured was available (88 out of the 160 soil samples). Other samples with lacking data for one or more variables were discarded from the analysis. The analysis was carried out on log-transformed data using a covariance matrix, implemented in the CANOCO software. To give the same weight to each variable, data were standardized and centered prior to the analysis, so that data average was zero with a variance of one. Differences between substrate types along the first four PCA axes were investigated by comparing scores with Kruskal-Wallis tests followed by multiple comparisons of mean ranks and Dunn-Šidák corrections. Differences between species and parcels were not investigated using component scores because there were too few records to reach significance.

## Results

We collected 173 termite samples and 116 associated substrate samples and 30 control soil samples. Termites belonged to 32 morphological species among which both *Longustitermes manni* and *Anoplotermes*-group sp E1 are species groups that include more than one species [32], but as they cannot be distinguished using morphological characters only, we retained both names, without additional splitting.

Kruskal-Wallis tests showed that all chemical variables significantly differed among substrate types, except for Cu^2+^, Zn^2+^, C/N and Al^3+^ for which no significant differences were detected (Table 1). Abandoned nests were the richest in nutrients and organic matter and differed from controls and bare soil samples for most of the measured variables (Table 1). A similar pattern was found for soil (palm) and soil (tree), which significantly differed from controls and bare soil for 8 and 6 measured variables, respectively (Table 1). Bare soil and controls never significantly differed from each other (Table 1). Bare soil had significantly lower Na^+^ concentration than soil (palm) and abandoned nests, and control had significantly lower Na^+^ concentration than abandoned nests, while other comparisons were not significant for Na^+^ (Table 1).

**Table 1:** Properties of soils associated with soil-feeding termites in four putative niche categories, with a non-parametric (Kruskal-Wallis) analysis of variance.

Variable	Control	Bare soil	Soil (tree)	Soil (palm)	Abandoned nest	H	n	p	p Dunn-Šidák correction
Ca (μg/g)	a (55.5)	a (44)	a,b (315)	a,b (114)	b (378)	29.04	144	***	***
Cd (μg/g)	b (0.08)	b (0.08)	a,b (0.17)	a (0.21)	a (0.32)	27.46	144	***	***
Cu (μg/g)	- (1.39)	- (0.92)	- (1.57)	- (2.43)	- (2.28)	8.21	144	0.084	-
Fe (μg/g)	c (398)	b,c (427)	a,b (1081)	a (1063)	a (1685)	37.99	144	***	***
K (μg/g)	c (56)	b,c (82)	a (197)	a,b (117)	a (243)	36.02	144	***	***
Mg (μg/g)	a (52)	a (50)	a,b (123)	a,b (97)	b (138)	19.98	144	***	*
Na (μg/g)	b (11)	b (13)	a,b (20)	a (31)	a (47)	26.84	144	***	***
Ni (μg/g)	- (0.27)	- (0.29)	- (0.51)	- (0.73)	- (1.27)	11.19	144	0.024	-
P (μg/g)	c (3.00)	b,c (2.82)	a,b,c (8.89)	a,b (8.64)	a (11.19)	23.8	144	***	**
Pb (μg/g)	b (4.2)	b (4.6)	a,b (5.9)	a (8.6)	a (8.1)	24.63	144	***	**
Zn (μg/g)	- (3.3)	- (2.5)	- (2.55)	- (4.1)	- (3.9)	6.53	144	0.163	-
MO (%)	c (13.8)	b,c (15.8)	a,b (28.4)	a (24.1)	a (29.8)	35.76	144	***	***
C (%)	a (3.9)	a,b (3.9)	b,c (7.6)	a,b,c (6.5)	c (10.9)	32.04	131	***	***
N (%)	a (0.26)	a (0.25)	b (0.44)	a,b (0.41)	b (0.62)	29.89	131	***	***
C/N	- (16.2)	- (16.2)	- (17)	- (16.7)	- (17.5)	6.49	131	0.166	-
pH_H2O_	- (4.2)	- (4.2)	- (4.2)	- (4)	- (4.4)	12.39	142	0.015	-
H^+^ (cmolc/kg)	- (0.32)	- (0.17)	- (0.43)	- (0.4)	- (0.2)	11.26	125	0.024	-
Al^3+^ (cmolc/kg)	- (0.4)	- (0.43)	- (0.57)	- (0.42)	- (0.58)	5.83	125	0.212	-
Ca^2+^ (cmolc/kg)	a,c (0.27)	a (0.21)	b,c (2.43)	a,b (0.37)	b (2.48)	29.92	123	***	***
Mg^2+^ (cmolc/kg)	a,c (0.38)	a (0.38)	b,c (1.22)	a,b (0.57)	b (1.19)	21.72	123	***	**
K^+^ (cmolc/kg)	a (0.13)	a (0.18)	b (0.44)	a,b (0.21)	b (0.62)	34.18	123	***	***
Na^+^ (cmolc/kg)	b,c (0.05)	c (0.05)	a,b,c (0.08)	a,b (0.10)	a (0.21)	25.26	123	***	***
CEC (cmolc/kg)	a (1.76)	a (1.57)	b (5.34)	a,b (2.31)	b (7.14)	32.21	123	***	***
pH_KCl_	- (4.1)	- (4.2)	- (4)	- (4)	- (4.2)	11.81	126	0.019	-
Sand (%)	b (49.1)	a,b (40.8)	a,b (27.3)	a (17.2)	a (25.0)	19.06	108	***	*
Silt (%)	b (15.6)	a,b (17.6)	a (36.4)	a,b (20.1)	a (27.5)	14.87	108	0.005	-
Clay (%)	a,b (39.2)	a,b (38.1)	b (33.2)	a (51.1)	a,b (37.9)	11.24	108	0.024	-

***, p<0.001

**, p<0.01

*, p<0.05.

Different letters indicate a significant difference (p<0.05). Numbers in parentheses are the median values.

**Fig 1:**
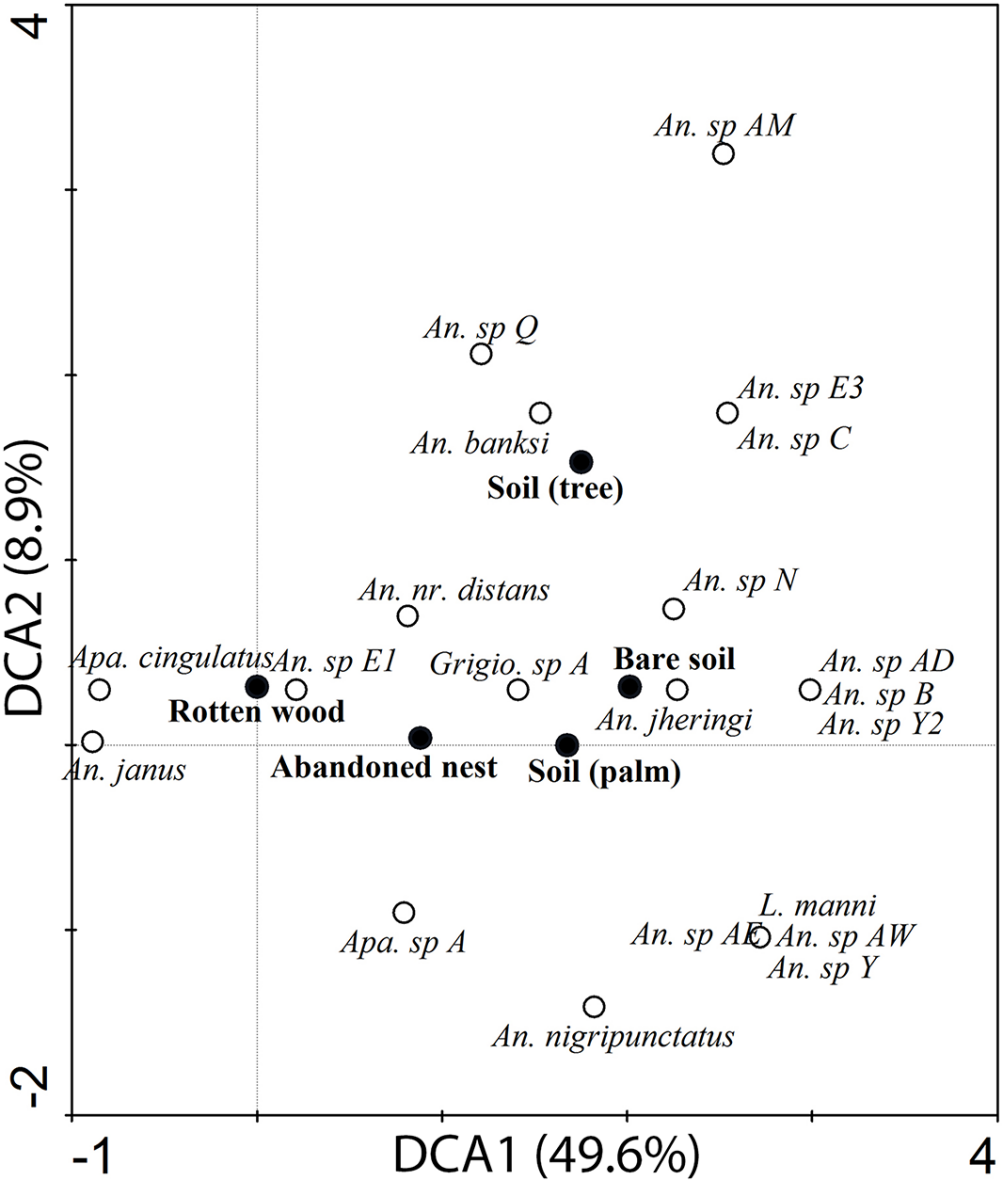
DCA bi-plot of termite species and substrate types. Solid symbols: substrate; open symbols: termite species.

**Fig 2:**
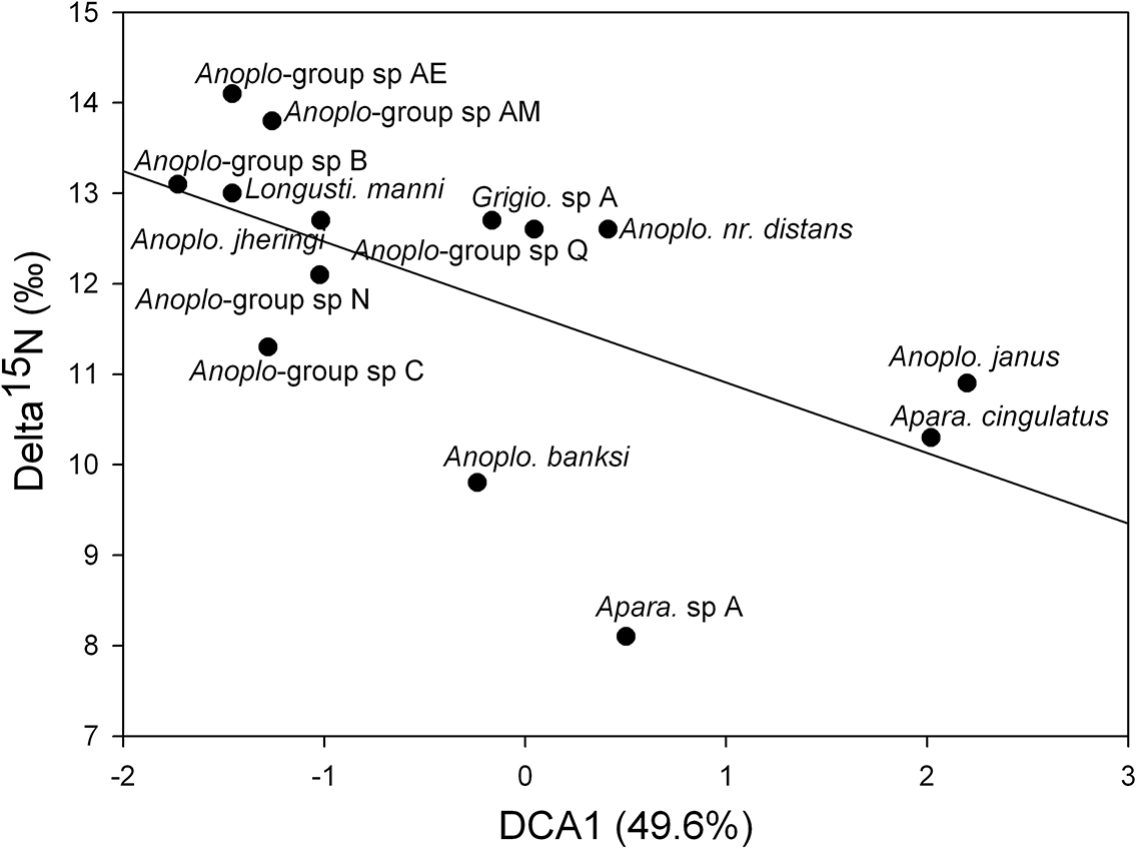
Scatter plot showing the correlation between species coordinates along the first axis of the DCA and δ^15^N isotopic values measured in [30].

The first and second axis of DCA computed on substrate types versus species matrix included 49.6 percent and 8.9 percent of the total variance (Fig 1). The gradient length was 3.93 for the first axis and 4.83 for the second axis (Fig 1). The first axis depicted substrates along a sequence beginning with “rotten wood” and ending with “bare soil”. Substrates did not order along a logical sequence along the second axis, which was characterized by a low eigenvalue and was thus likely to contain only minor information. The species coordinates along the first axis of the DCA, were significantly correlated with δ^15^N isotopic values measured in [30] (Pearson correlation: r = -0.588, t = -2.516, df = 12, p = 0.027) (Fig 2), values that reflect species position along the wood-soil decomposition gradient.

Kruskal-Wallis tests independently comparing the 27 soil parameters of bare soil associated with the six species for which a minimum of four soil samples were collected (*Anoplotermes*-group sp B, sp E1, sp AD, sp N, *Anoplotermes nr*. *distans* and *Longustitermes manni*) showed no differences for any variable except for Cu (H_5,30_ = 13.06, p = 0.023, p (Dunn-Šidák corrected) > 0.05), organic matter content (H_5,30_ = 12.75, p = 0.026, p (Dunn-Šidák corrected) > 0.05) and silt proportion (H_5,24_ = 13.24, p = 0.021, p (Dunn-Šidák corrected) > 0.05), when p values were not corrected for the effect of multiple comparisons. None of the Dunn-Šidák corrected p values were significant.

**Fig 3:**
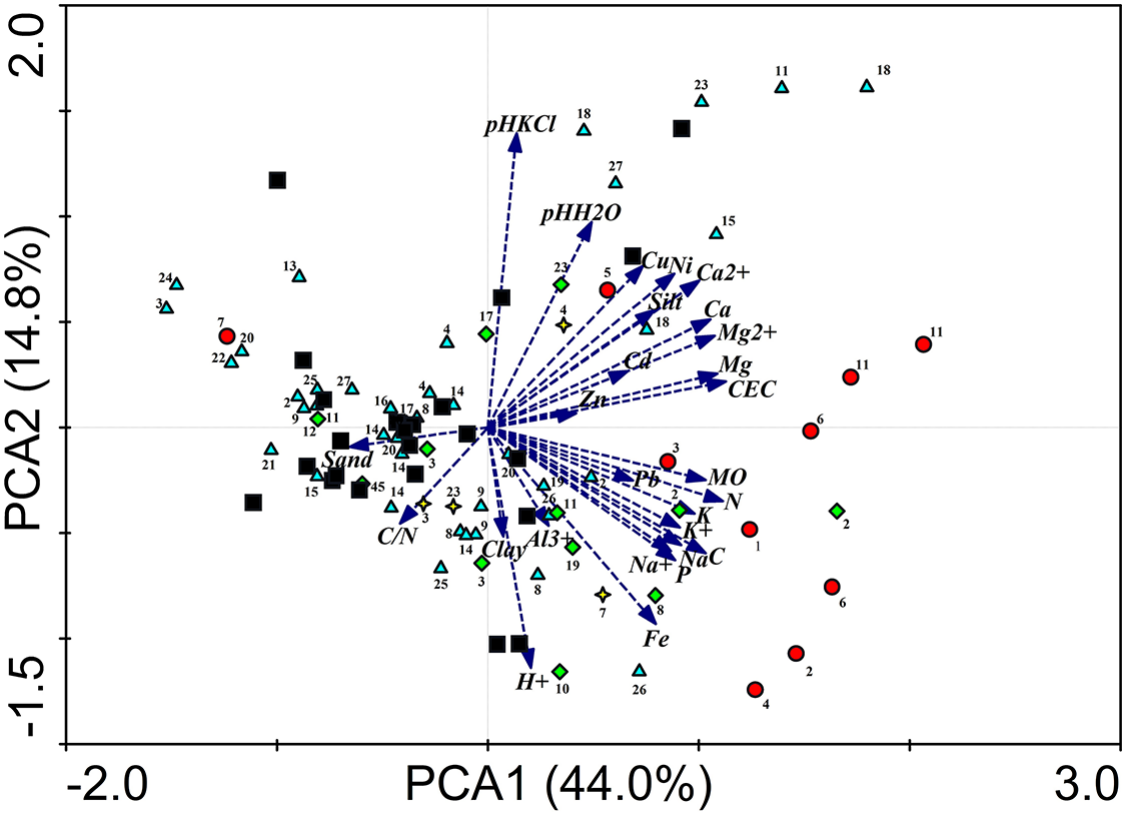
PCA bi-plot of termite samples and environmental variables depicting the first and second axes. Black filled squares: controls; light blue-filled triangles: termites sampled in soil; red-filled circles: termites sampled in abandoned nests; yellow-filled stars: termites sampled at tree basis; green-filled diamond: termites sampled at the basis of palmtree. 1. *A*. *banksi*; 2. *A*. *nigripunctatus*; 3. *A*. *jheringi*; 4. *A*. nr *distans*; 5. *Apara*. *cingulatus*, 6. *Apara*. sp A; 7. *Grigio*. sp A; 8. *Longusti*. *manni*; 9. *A*. sp B; 10. *A*. sp C, 11. *A*. sp E1; 12. *A*. sp E3; 13. *A*. sp I; 14. *A*. sp N; 15. *A*. sp S; 16. *A*. sp T; 17. *A*. sp Y; 18. *A*. sp Y2; 19. *A*. sp AB; 20. *A*. sp AD; 21. *A*. sp AE; 22. *A*. sp AF; 23. *A*. sp AM; 24. *A*. sp AN; 25. *A*. sp AP; 26. *A*. sp AW; 27. Unidentified species.

PCA reduced the 27 variables to three main axes capturing 67% of the total variance. The first and second axis of PCA captured 44.0 percent and 14.8 percent of the total variance, respectively (Fig 3). The first axis was highly correlated with most of the 27 measured soil parameters and was best explained by a gradient of organic matter and mineral element abundance. Samples did not order randomly along this axis concerning substrate types (Kruskal Wallis: H = 20.02, N = 4, 89, p < 0.001, p (Dunn-Šidák corrected) < 0.01), abandoned nests having higher scores and significantly differing from controls (Z = 3.98, p < 0.001) and from bare soils (Z = 3.73, p = 0.002). The second axis mostly represented a pH gradient, but substrate types did not differ in their score along it (Kruskal Wallis: H = 7.33, N = 4, 89, p = 0.120). The third axis, capturing 9.2% of the total variance, was a particle size gradient (clay, silt and sand) (S1 Fig), with significant differences among substrate types (Kruskal Wallis: H = 16.45, N = 4, 89, p = 0.0025, p (Dunn-Šidák corrected) < 0.01), soil (palm) differing from bare soils (Z = 2.98, p = 0.029), abandoned nests (Z = 3.83, p = 0.001) and controls (Z = 2.85, p = 0.044).

## Discussion

### Do soil-feeding termite species differ in the foraging substrate?

Our results suggest that the niche partitioning in Neotropical soldierless termites, as previously revealed by δ^15^N isotopic analyses [30], is caused by preferential feeding on specific substrates and that further subcategories within a foraging substrate cannot be recognized. Examples of species-specialization include *Aparatermes cingulatus* and *Ap*. sp A, which are characterized by a low score along the first axis of the DCA (Fig 1), indicating that they feed on substrates rich in organic matter such as rotten wood or abandoned nests; other species, such as *Longustitermes manni*, *An*. *jheringi* or *An*. sp AD, obtained high scores along the first axis of the DCA, showing their association with soil poor in organic matter content. Interestingly, species position along the first axis, reflecting their type of foraging substrate, was positively correlated with δ^15^N isotopic values. A factor explaining interspecific differences previously measured with δ^15^N isotope ratios could therefore be species specialization toward a particular substrate type, which was not taken into account previously [30]. By contrast, no differences were observed among samples of bare soil exploited by six different species, although including additional species could give different results.

The fact that several species of the *Anoplotermes*-group feed on the same type of soil may suggest they share the same feeding niche, which would contrast with other soil arthropods such as oribatid mites [7, 8] and springtails [9, 10]. In this study, the substrates we analysed contained at least tens of termites, and therefore represented, with few possible exceptions, foraging sites rather than galleries connecting the nest to foraging area; the collected soil samples thus most likely represented the actual substrate termites fed on. However, the scale at which our samples were collected, namely spoonfuls of soil around termite foraging parties, may not match the scale at which individual termites actually select food particles.

The suggested absence of feeding niche differentiation among members of the *Anoplotermes*-group feeding on bare soil also seems surprising because of the highly variable morphology of the worker digestive tract in this group. One structure of special interest is the enteric valve, which often bears a sclerotized armature whose shape is highly diverse among species [32, 35, 36, 37]. The anatomy of the enteric valve and other digestive tract structures undoubtedly plays a role in digestion, but it might not be as appropriate to distinct feeding groups as previously suggested [25].

Although soil-feeding termites are frequently found within items of dead wood, this substrate was not analysed in the present work, because it is fully organic and cannot be compared to soil in many aspects such as its texture. *Anoplotermes*-group termites occurring in the wood seem to feed exclusively on the already decomposed fraction of the wood as suggested by stable isotope data [26, 30].

### Do soil-feeding termites select their foraging sites to enhance nutrients intake?

We measured 27 variables to determine the influence of soil composition and structure on termite communities. The 27 variables were highly correlated together as shown by a PCA whose first three axes explained 67% of the variance (Fig 3, S1 Fig). Rich soil patches have consistently high content in all nutrients and poor soil patches are poor in all nutrients (Fig 3, S1 Fig), suggesting that targeting rich soil patches would enhance intake of all nutrients simultaneously. However, we found no evidence that termites feeding in bare soil target nutrient rich patches.

Palm tree crowns work as a funnel gathering large amounts of leaf litter which accumulates on the ground underneath and is degraded by decomposers. Similarly, abandoned termite nests are commonly inhabited by secondary occupants, which use them as shelter and food source [38]. In agreement with previous studies [39, 40], our results show that abandoned nests are richer in organic matter and nutrients than bare soil, but not than soil (palm) and soil (tree), which are of comparable richness in organic matter and nutrients (Table 1, Fig 3, S1 Fig). High nutrient content makes nests attractive to a large number of termite species among which some, like *Inquilinitermes* spp., are obligatorily associated to the nest of other species.

Many species were also collected only from the bare soil and never occurred in richer substrates. We tested whether bare soils (with termites) were in nutrient content richer compared to controls (bare soils without termites), but our results did not reveal any significant difference. Soil-feeding termites thus did not select richer soil patches. As also the case for tropical forest ants, soil structure and composition therefore seem to be unimportant for the structuring termite communities [41]. Previous studies found that sodium enhances litter termite abundance in some areas [15], but no such influence was detected here. One possible explanation is that sodium and other nutrients are available in sufficient amounts, and therefore do not limit population size in the Nouragues reserve, which is located about 100 km away from the sea and therefore receives sea-water aerosols [15]. Additionally, many parameters differ between our study and Kaspari et al. [15]'s, including the sampling layer (soil vs. litter) and treatments (random sampling in natural conditions vs. high experimental enrichment of small patches), making comparisons difficult. Overall, our results indicate that unlike abandoned nests or soil (palm) which are targeted by many species, termites foraging on bare soil do not preferentially select richer soil patches and are indifferent to organic matter and mineral content, at least within the range of variation naturally observed in the Nouragues forest.

Our results indicate that niche partitioning occurs among the so-called “soil-feeding” termites through the selection of species-specific feeding substrates (e.g., organic matter-rich substrates such as abandoned nests or soil at the base of palms). However, any further subcategories among *Anoplotermes*-group species feeding on soil *sensu stricto* could not be recognized, which suggests that these species do not select foraging patches according to their texture or composition. Additional studies are needed to test whether or not these species differ by selection of food items at a higher scale of resolution. If they occupy the same trophic niche, a further question is whether other ecological or behavioural features (nest site, colony size, reproductive strategies) contribute to niche separation. Alternatively, a broad overlap of niches would suggest, according to neutral theory, the importance of random drift effects in shaping the termite community.

## Supporting Information

S1 Fig PCA bi-plot of termite samples and environmental variables depicting the first and third axes. Black filled squares: controls; light blue-filled triangles: termites sampled in soil; red-filled circles: termites sampled in abandoned nests; yellow-filled stars: termites sampled at tree basis; green-filled diamond: termites sampled at the basis of palmtree. 1. *A*. *banksi*; 2. *A*. *nigripunctatus*; 3. *A*. *jheringi*; 4. *A*. nr *distans*; 5. *Apara*. *cingulatus*, 6. *Apara*. sp A; 7. *Grigio*. sp A; 8. *Longusti*. *manni*; 9. *A*. sp B; 10. *A*. sp C, 11. *A*. sp E1; 12. *A*. sp E3; 13. *A*. sp I; 14. *A*. sp N; 15. *A*. sp S; 16. *A*. sp T; 17. *A*. sp Y; 18. *A*. sp Y2; 19. *A*. sp AB; 20. *A*. sp AD; 21. *A*. sp AE; 22. *A*. sp AF; 23. *A*. sp AM; 24. *A*. sp AN; 25. *A*. sp AP; 26. *A*. sp AW; 27. Unidentified species.
(TIF)

S1 Table Raw data for the 148 samples analysed in this study. (XLSX)
