# Correction: Influence of Soil Properties on Soldierless Termite Distribution

**DOI:** 10.1371/journal.pone.0143776

**Published:** 2015-11-23

**Authors:** Thomas Bourguignon, Thomas Drouet, Jan Šobotník, Robert Hanus, Yves Roisin

There are errors in the Funding section; the Internal Grant Agency of Faculty of Forestry and Wood Sciences (Specific research of the Czech University of Life Sciences) (project: A08/14) did not provide any financial support for this study. Instead, support was received from the projects CIGA No. 20154318 and IGA No. B03/15 (Czech University of Life Sciences, Prague).

The complete, correct funding statement should read as follows: Financial support was provided by the Belgian FRIA (fellowship to TB), the French CNRS (Nouragues grant), the Institute of Organic Chemistry and Biochemistry (RVO: 61388963), and the projects CIGA No. 20154318 and IGA No. B03/15 (Czech University of Life Sciences, Prague). TB was supported by the University of Sydney through a postdoctoral fellowship.

## References

[1] Bourguignon T, Drouet T, Šobotník J, Hanus R, Roisin Y (2015) Influence of Soil Properties on Soldierless Termite Distribution. PLoS ONE 10(8): e0135341 doi:10.1371/journal.pone.0135341 26270057 10.1371/journal.pone.0135341 PMC4536034

