# Supplementary figures and images for: Influence of Soil Properties on Soldierless Termite Distribution
Source: PLoS One. 2015 Aug 13;10(8):e0135341. doi: 10.1371/journal.pone.0135341 (PMC4536034; doi:10.1371/journal.pone.0135341)

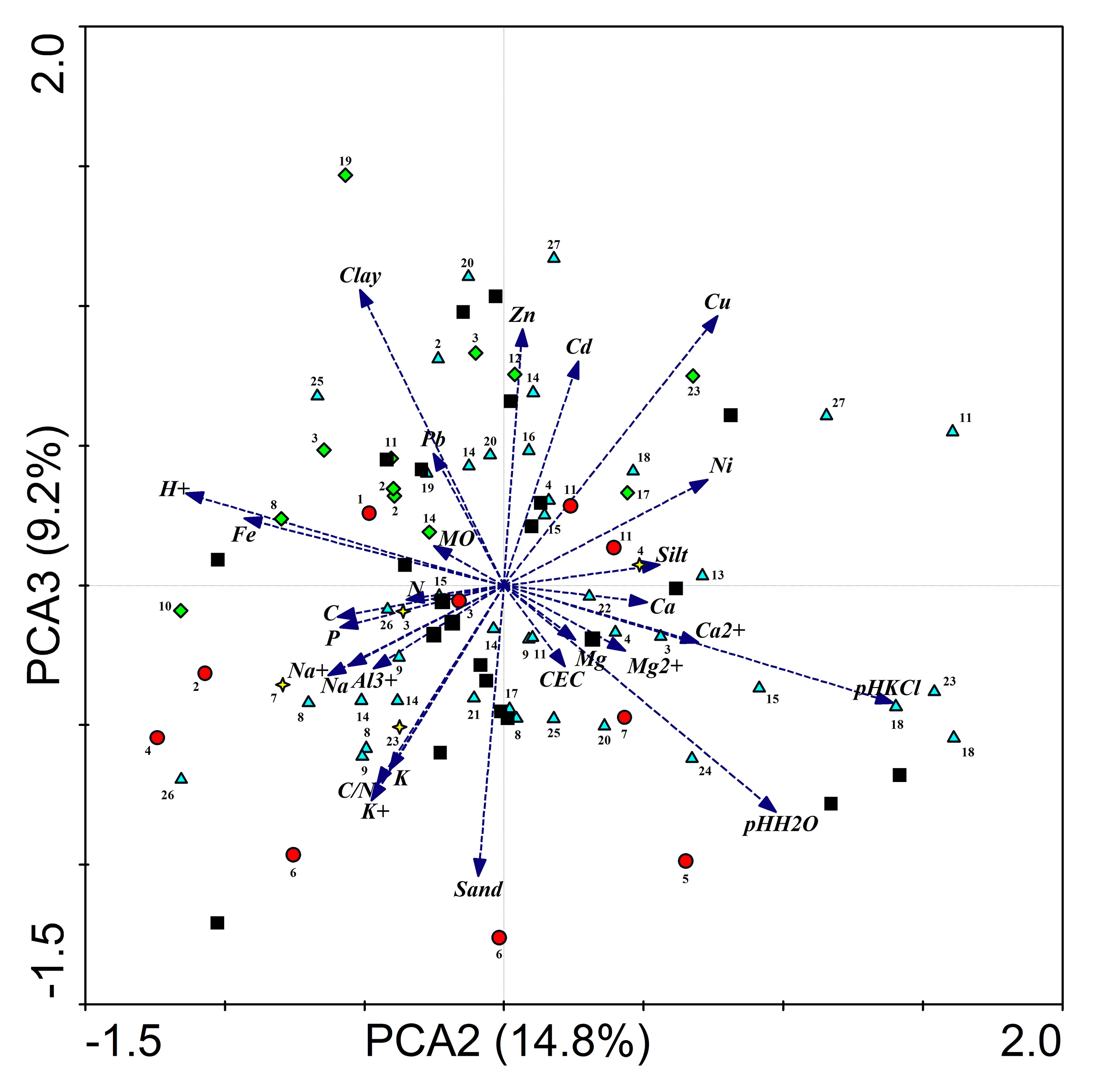

Supplement: S1 Fig — Black filled squares: controls; light blue-filled triangles: termites sampled in soil; red-filled circles: termites sampled in abandoned nests; yellow-filled stars: termites sampled at tree basis; green-filled diamond: termites sampled at the basis of palmtree. 1. A. banksi; 2. A. nigripunctatus; 3. A. jheringi; 4. A. nr distans; 5. Apara. cingulatus, 6. Apara. sp A; 7. Grigio. sp A; 8. Longusti. manni; 9. A. sp B; 10. A. sp C, 11. A. sp E1; 12. A. sp E3; 13. A. sp I; 14. A. sp N; 15. A. sp S; 16. A. sp T; 17. A. sp Y; 18. A. sp Y2; 19. A. sp AB; 20. A. sp AD; 21. A. sp AE; 22. A. sp AF; 23. A. sp AM; 24. A. sp AN; 25. A. sp AP; 26. A. sp AW; 27. Unidentified species. (TIF) [file pone.0135341.s001.tif]
